# Oocyte maturation with royal jelly increases embryo development and reduces apoptosis in goats

**DOI:** 10.21451/1984-3143-2017-AR986

**Published:** 2018-08-16

**Authors:** Arash Veshkini, Abdollah Mohammadi-Sangcheshmeh, Nasser Ghanem, Amir Hossein Abazari-kia, Elmira Mottaghi, Reza Kamaledini, Hamid Deldar, Irfan Ozturk, Eduardo Leite Gastal

**Affiliations:** 1 Department of Transgenic Animal Science, Stem Cell Technology Research Center, Tehran, Iran.; 2 Department of Animal and Poultry Science, College of Aburaihan, University of Tehran, Pakdasht, Tehran, Iran.; 3 Department of Animal Production, Faculty of Agriculture, Cairo University, Giza, Egypt.; 4 Department of Animal Science and Technology, Faculty of Agriculture and Natural Resources, Tehran Science and Research Branch, Islamic Azad University, Tehran, Iran.; 5 Department of Animal Science, College of Animal Science and Fisheries, Sari Agricultural Sciences and Natural Resources University, Sari, Iran.; 6 Department of Biometry Science, Faculty of Agriculture, Harran University, Sanliurfa, Turkey.; 7 Department of Animal Science, Food and Nutrition, Southern Illinois University, Carbondale, IL, USA.

**Keywords:** embryo, gene expression, *in vitro* fertilization, oocyte, royal jelly

## Abstract

Royal jelly (RJ) was supplemented to goat oocyte *in vitro* maturation (IVM) medium at three different concentrations (2.5, 5, and 10 mg/ml). Maturation rate, embryo cleavage, and blastocyst rate were recorded. Gene expression of apoptosis-related transcripts was investigated in matured oocytes. Percentage of oocytes that reached MII-stage was increased in RJ-treated groups compared to the control group. Glutathione (GSH) content of mature oocytes was enhanced when RJ was added to IVM medium at any supplementation compared with control. Percentage of cleaved embryos and blastocysts was higher in the RJ-treated groups at a concentration of 5 than in the 2.5 mg/ml and control group. Total number of cells per blastocyst was not different in the control and RJ-treated group at 5 mg/ml. However, number of apoptotic cells per blastocyst was higher in the control group than in the RJ-treated group at 5 mg/ml. Expression profile of *Bax*, and *p53* was down-regulated while *Bcl-2* was up-regulated in oocytes treated with RJ at 5 and 10 mg/ml compared with the control group. Addition of RJ at concentrations of 5 mg/ml improved embryo production through increasing maturation rate. RJ seems to improve the IVM microenvironment by reducing expression of genes inducing apoptosis, enhancing GSH content, and reducing incidence of apoptosis in blastocysts.

## Introduction

The efficiency of assisted reproductive technologies (ARTs) like *in vitro* embryo production, intracytoplasmic sperm injection (ICSI), and somatic cell nuclear transfer (SCNT) is completely dependent on the production of higher numbers of transferable embryos of good quality. Indeed, *in vitro* maturation (IVM) of cumulus-oocytes complexes (COCs) is a key step in improving the outcome of ARTs. Well-established culture systems used for IVM have substantially influenced the maturation rate, *in vitro* fertilization (IVF) rate, and subsequent embryonic development rate ( Brackett and Zuelke, 1993 ; Cox and Alfaro, 2007 ; Combelles *et al.,* 2009 ; Souza-Fabjan *et al*., 2014 ; Fakruzzaman *et al.,* 2015 ). Under *in vivo* conditions, COCs are provided with all essential minerals, growth factors, proteins, and natural buffering agents that cannot be mimicked totally under *in vitro* counterparts. However, developmental capabilities of COCs can be improved by supplementation of various hormones, growth factors, serum, cells, follicular fluid, and other substances added to the maturation media ( Romero-Arredondo and Seidel, 1996 ; Choi *et al.,* 2001 ; Zhou *et al*., 2016 ; Soto-Heras *et al*., 2018 ). In this context, the blastocyst rate was improved, reaching up to 70% when COCs were matured *in vivo*, while this rate dropped to 30-35% under IVM conditions ( Wrenzycki and Stinshoff, 2013 ).

Substantial enhancement of early embryonic development has been reported ( Albuz *et al.,* 2010 ) when the IVM system was improved in different mammalian species. For example, the blastocyst development rate was increased in mice (86%) and cattle (69%) by applying simulated physiologic oocyte maturation. Furthermore, the implantation rate (53%) and fetal yield (26%) were also improved in mice ( Albuz *et al.,* 2010 ). From a practical point of view, improving IVM conditions holds much promise in cattle and human ARTs ( Edward, 2007 ). Although many factors have shown substantial contribution to *in vitro* embryo production (IVEP), maintenance of adequate balance between production and scavenging of oxidative stress is of great importance ( Guerin *et al.,* 2001 ). Generally, COCs are negatively affected by increased oxidative stress during IVM due to higher reactive oxygen species (ROS) than that under an *in vivo* environment. Under *in vivo* conditions, oocytes and embryos are able to resist oxidative stress by antioxidants, which are present in follicular fluid ( Oyawoye *et al.,* 2003 ) or produced by embryos themselves in the oviduct ( Gardiner and Reed, 1995 ). In this regard, royal jelly has been described as an effective antioxidant ( Bărnuţiu *et al.,* 2011 ). Royal jelly is a viscous secretion of both hypopharyngeal and mandibular glands of young worker bees that is used to feed the queen honeybee throughout the larval period ( Viuda-Martos *et al.,* 2008 ; Isidorova *et al.,* 2009 ). It contains the following: water (50-60%), proteins (18%), carbohydrates (15%), lipids (3-6%), mineral salts (1.5%), and vitamins, in addition to various bioactive substances ( Howe *et al*., 1985 ; Boselli *et al*., 2003 ; Nagai and Inoue 2004 ; Kodai *et al*., 2007 ; Tamura *et al*., 2009 ). The antioxidative capacity of royal jelly was shown to be effective in protecting female and male gametes ( Guo *et al.,* 2009 ) and cryopreserved semen ( Moradi *et al.,* 2013 ; Shahzad *et al.,* 2016 ).

The current study is the first to test if the supplementation of royal jelly as an antioxidant agent during IVM culture of goat COCs could be beneficial. After culture, nuclear maturation, glutathione content, preimplantation development, and the expression profile of apoptosis-related transcripts were examined.

## Materials and Methods

### Chemicals and media

Unless otherwise mentioned, all the chemicals, reagents, media, and constituents used for media preparation were purchased from Sigma-Aldrich Chemicals, USA. Capsulated pure royal jelly was provided by Natural Life^TM^ (Brookvale, NSW, Australia). According to manufacturer’s reports, the chemical composition was water 1.6% (w/w), proteins 40.1% (w/w), carbohydrates 47.3% (w/w), lipids 7.9% (w/w), ashes 3.1% (w/w), sodium 36.3 mg/100 g, phosphorus 672 mg/100 g, calcium 19.5 mg/100 g, magnesium 90.9 mg/100 g, zinc 6.8 mg/100 g, iron 3.0 mg/100 g, and free amino acids 1583 mg/100 g.

### COC collection and IVM

Goat ovaries were collected from a local slaughterhouse and transported in physiological saline containing antibiotics (100 IU/ml penicillin and 100 mg/ml streptomycin) at 35°C to the lab within 2-3 h. COCs were collected by slicing the ovaries in HEPES-buffered synthetic oviductal fluid (HSOF). The COCs were morphologically selected based on the number of cumulus cell layers and cytoplasm homogeneity ( Mohammadi-Sangcheshmeh *et al.,* 2012 ). The collected COCs were washed three times in maturation medium (TCM-199 supplemented with 2 mM l-glutamine, 10% fetal bovine serum, 5.5 mg/ml sodium pyruvate, 25 μg/ml gentamycin sulphate, 5.0 μg/ml LH, 0.5 μg/ml FSH, and 1 μg/ml estradiol). IVM was performed in 50 μl maturation medium droplets under mineral oil for 24 h at 39°C and 5% CO_2_ in air.

### Evaluation of cumulus expansion

COCs from all treatment groups were evaluated at the end of IVM to assess cumulus cell expansion. Briefly, cumulus expansion was evaluated by a subjective scoring system ( Lorenzo *et al.,* 1994 ), which considered no detectable response set as (0) value and highest degree of expansion as (+++).

### Nuclear chromatin evaluation

For nuclear maturation assessment, cumulus cells were removed from COCs by pipetting in presence of hyaluronidase (200 U/ml). Denuded oocytes were fixed in 4% (v/v) paraformaldehyde in PBS. Oocytes were stained with 2.5 mg/ml Hoechst 33258 in 3:1 (v/v) glycerol/PBS. Oocytes were then evaluated in relation to their meiotic stage and classified as germinal vesicle (GV); metaphase I (MI), including metaphase I, anaphase I, and telophase I; metaphase II (MII); and degenerated.

### Measurement of intracellular GSH content

Mature COCs were denuded directly after IVM and incubated in Tyrode’s medium plus 5 mg/ml polyvinyl alcohol containing 10 mM 4-chloromethyl-6,8-difluoro-7-hydroxycoumarin (Cell tracker blue; CMF_2_HC; Invitrogen Corporation, Carlsbad, CA, USA) for 30 min ( Abazari-Kia *et al.,* 2015 ). After staining, oocytes were washed three times in mPBS, placed into 10 μl droplets, observed under an epifluorescence microscope (Nikon, Tokyo, Japan) with UV filters, and then all fluorescent images were recorded as graphic files. The fluorescence intensity of each mature oocyte was analyzed by ImageJ software ( http://rsb.info.nih.gov/ij ).

### IVF and embryo culture

Following IVM and cumulus expansion evaluation, COCs were washed twice in HSOF and once in IVF medium [SOF supplemented with 4 IU/ml heparin, PHE (20 mM penicillamine, 10 mM hypotaurine, 1 mM epinephrine), and 2% (v/v) sheep serum], then placed in 50 μl drops of IVF medium overlaid with mineral oil. Frozen semen from a goat buck that had been evaluated before for IVEP was used for IVF. A single straw of frozen semen was thawed at 37°C for 30 sec and sperms were washed at 500 g for 10 min, twice with Sperm Tyrode’s Albumin Lactate Pyruvate medium (Sperm-TALP) containing 10 μg/ml heparin, 2.2 mg/ml sodium pyruvate and bovine serum albumin (BSA) F-V (6 mg/ml) + 50 μg/ml gentamycin. After washing, a sperm pellet was suspended in 0.5 ml of fresh Fert-TALP medium supplemented with 6 mg/ml BSA (fatty acid free) + 10 μg/ml heparin + 3 μl PHE and 50 μg/ml gentamycin. Sperm concentration was adjusted to 2 × 10^6^ spermatozoa/ml. The washed suspended sperms were incubated with mature COCs for 18 h under 5% CO2 in humidified air at 39°C.

The fertilized COCs were denuded by repeat pipetting and thoroughly washed three times in HSOF. The presumptive zygotes were washed in SOFaa (2% BM Essential amino acids, 1% MEM-nonessential amino acids) and supplemented with 8 mg/ml bovine serum albumin, 1 mM glutamine, 0.34 mM tri-sodium citrate, and 2.77 mM myoinositol. The presumptive zygotes were cultured in groups of 15 to 20 in 50 µl droplets of the culture medium (SOFaa) and covered with mineral oil at 39°C under 5% CO2 in humidified air until day 8. The stage of embryonic development was evaluated at day 3 and 8 post-fertilization, and the medium replacement was performed every 48 h.

### Total cell count and number of apoptotic cells assay

The terminal deoxynucleotidyl transferase dUTP nick end labeling (TUNEL) assay was performed using an In Situ Cell Death Detection Kit according to the manufacturer’s instructions (Fluorescein; Roche Diagnostics Corp., Indianapolis, USA). Day 8 blastocysts from the control and 5 mg/ml royal jelly-treated groups were washed twice in PBS (Ca^2+^ and Mg^ 2+^ free) supplemented with 0.2% polyvinylpyrrolidone (PBS-PVP). Embryos were then fixed in 4% paraformaldehyde in PBS-PVP and kept at 4°C until assay was done. Fixed embryos were first washed twice in PBS-PVP then treated with 0.3% Triton X-100 in PBS-PVP for 30 min at 37°C for permeabilization. Embryos were washed twice in PVP-PBS and incubated in the dark with TUNEL reaction mixture for 1 h at 37°C. Subsequently, embryos were washed three times in PBS-PVP for 10 min each and stained with Hoechst 33342 (10 μg/ml). After staining, embryos were placed onto a glass slide and examined using an epifluorescence microscope (Nikon, Tokyo, Japan).

### RNA isolation

Total RNA was extracted using PicoPure^TM^ RNA isolation kit (MDS Analytical Technologies GmbH, Ismaning, Germany) according to manufacturer’s instructions. Mature oocytes from each group were incubated in 100 µl extraction buffer at 42°C for 30 min to release RNA. The lysis of each group was loaded onto a pre-conditioned purification column and centrifuged to allow for the RNA to bind to the spin column. In-column DNA digestion was carried out using RNase-free DNase (Qiagen GmbH, Hilden, Germany). The column was washed twice with washing buffer and finally eluted with 12 µl RNase free water. Nanodrop 1000 Spectrophotometer (Thermo Fisher Scientific) was used to measure RNA concentration and evaluate the quality.

The synthesis of cDNA from all samples was performed using reverse transcription kit (Invitrogen, Karlsruhe, Germany). In addition, 1 μl oligo dT23 (2.5 μM) primer and 1 μl random hexamer primer (60 μm) were added to a 10 μl mRNA sample and the mixture was incubated for 3 min at 70°C and cooled on ice. Eight microliters of the master mix containing 4 μl of 5x first strand buffer, 2 μl of 0.1 M DTT, 1 μl of dNTP (10 pmol/μl), and 0.3 μl of RNase inhibitor and 0.7 μl of SuperScript IITM reverse transcriptase (200 unit/μl) was added to the mixture and incubated for 90 min at 42°C, followed by heat inactivation for 15 min at 70°C. The synthesized cDNA was stored at -20°C for further use.

### Quantitative real-time PCR analysis

Primers used for quantitative Real-time PCR were designed using Primer3 Express version 4.0.0 software ( http://primer3.wi.mit.edu// ) based on the gene sequences available in NCBI GenBank ( Table 1 ). Bovine YWHAZ gene was used as an endogenous control, and run in separate wells using ABI PRISM® 7000 instrument (Applied Biosystems). All PCR runs were done in a total volume of 20 µl containing 10 µl of 2.5 X RealMasterMix/20x SYBR (Eppendorf, Hamburg, Germany). Samples were run in duplicate to maximize accuracy of real-time results. The thermal cycling was set as 10 sec at 50°C, 10 min at 95°C, 45 cycles of 15 sec at 95°C and 60 sec at 60°C. The dissociation curve was generated by starting the fluorescence acquisition at 60°C and taking measurements every 7 sec until the temperature reached 95°C. Gene expression analysis was performed using ΔΔ C(t) method after normalization of the transcript abundance of each target gene relative to that of the *YWHAZ.*

**Table 1:** Details of primers used for real-time PCR quantitative analysis.

Gene name	GenBank accession number	Primer sequences	Annealing temperature (°C)	Product size (bp)
*Bax*	NM_173894.1	F:5′-GCATCCACCAAGAAGCTGAG-3′ R:5′-CCGCCACTCGGAAAAAGAC-3′	61	130
*Bcl-2*	NM_001166486.1	F:5′-ATGTGTGTGGAGAGCGTCA-3′ R:5′-AGAGACAGCCAGGAGAAATC-3′	60	182
*p53*	NM_174201.2	F:5′-AGGGGAAAGCAGGGCTCACTCT-3′ R:5′-GGGATATGGGTGGGGATGTCAA-3′	60	151
*YWHAZ*	NM_174814.2	F:5′-GAAGAGTCCTACAAAGACAGCACGC-3′ R:5′-AATTTTCCCCTCCTTCTCCTGC-3′	60	115

F, forward; PCR, polymerase chain reaction; R, reverse.

### Statistical analyses

The data of gene expression profile was analyzed using General Linear Model (GLM) of the Statistical Analysis System (SAS) software program 8.0 (SAS Institute Inc., NC, USA). Mean values were considered significant at P < 0.05 and after tested using ANOVA followed by a multiple pair wise comparison of t-test.

### Experimental design

Cumulus-oocyte complexes recovered from slaughterhouse ovaries were allocated into three groups during IVM in presence of RJ with three different concentrations (2.5, 5, and 10 mg/ml). Following IVM, cumulus expansion score and nuclear maturation rate were checked to evaluate the effect of treatment on IVM. The presumed zygotes were then cultured without any supplementation to follow up embryo development until blastocyst formation at day 8. Based on the result of nuclear maturation and blastocyst rates, RJ concentration of 5 mg/ml was selected for investigating embryo quality (total cell number and apoptotic cell number). The beneficial effect of RJ supplementation on goat COCs during IVM was further studied at cytoplasmic and molecular levels. Therefore, GSH content was measured in all treatment groups. In addition, gene expression profile of apoptosis-related transcripts was performed using quantitative real-time PCR.

## Results

### Cumulus expansion

**Figure 1:**
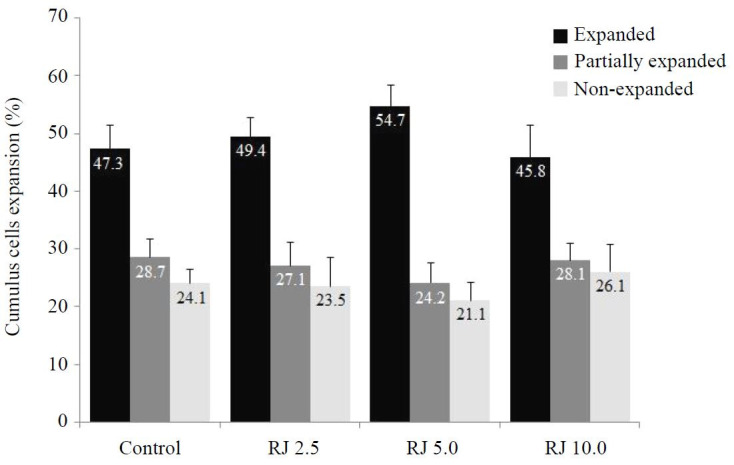
Percentages of cumulus expansion score (fully expanded, partially expanded, and non-expanded) of COCs treated with different concentrations of royal jelly during IVM. ^a,b,c,d^ Bars with uncommon superscripts are different (P < 0.05).

The data of COCs that attained expansion is shown ( Fig. 1 ). No difference in cumulus expansion score was detected among the different royal jelly-treated groups and the control. Although not significant (P > 0.05), the percentage of fully expanded COCs increased when royal jelly was supplemented to IVM medium at a concentration of 5 mg/ml (54.7%) compared with 2.5 mg/ml (49.4%), 10 mg/ml (45.8%), and control (47.3%) groups.

### Nuclear maturation

The percentage of oocytes that reached MII-stage increased (P < 0.05) steadily with increasing concentrations of royal jelly during IVM groups ( Table 2 ). In addition, the percentage of MII-stage oocytes was higher at 10 mg/ml (88.0% ± 1.7), 5 mg/ml (80.9% ± 0.6), and 2.5 mg/ml (71.4% ± 2.3) than in the control (60.0% ± 1.7) group.

**Table 2:** Nuclear status of goat oocytes treated with different concentrations of royal jelly after 24 h of *in vitro* maturation (cumulative results of five replicates).

	Nuclear status (mean ± SEM)				
Group	Oocytes (n)	GV (%)	GVBD (%)	MI (%)	MII (%)
Control	110	14.5 ± 0.6^a^	10.9 ± 0.0^a^	14.5 ± 1.1^a^	60.0 ± 1.7^c^
RJ 2.5	168	5.9 ± 0.3^b^	8.3 ± 0.6^ab^	14.3 ± 0.6^ab^	71.4 ± 2.3^b^
RJ 5.0	126	3.2 ± 0.6^c^	6.3 ± 0.3^ab^	9.5 ± 0.0^b^	80.9 ± 0.6^ab^
RJ 10.0	134	1.5 ± 0.0^c^	3.0 ± 0.6^b^	7.5 ± 1.1^b^	88.0 ± 1.7^a^

RJ 2.5: Control maturation medium supplemented with 2.5 mg/ml royal jelly; RJ 5.0: Control maturation medium supplemented with 5.0 mg/ml royal jelly; RJ 10.0: Control maturation medium supplemented with 10.0 mg/ml royal jelly; GV: Germinal vesicle; GVBD: Germinal vesicle break down; MI: Metaphase I; MII: Metaphase II. ^a,b,c^ Within columns, values with uncommon superscripts differ (P < 0.05).

### Glutathione content

**Figure 2:**
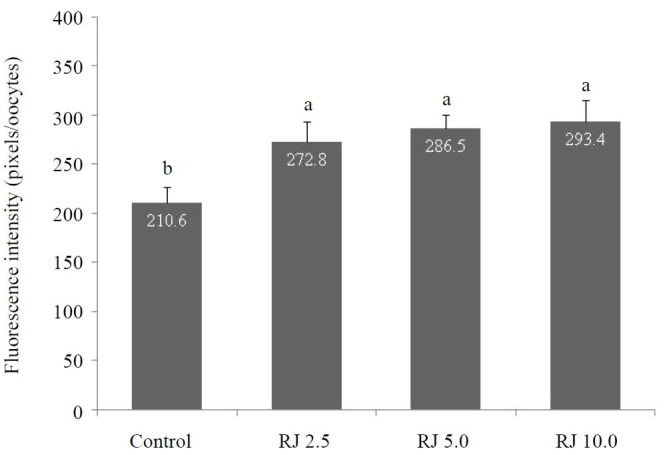
Glutathione content of oocytes treated with different concentrations of royal jelly during IVM. ^a,b,c,d^ Bars with uncommon superscripts are different (P < 0.05).

**Figure 3:**
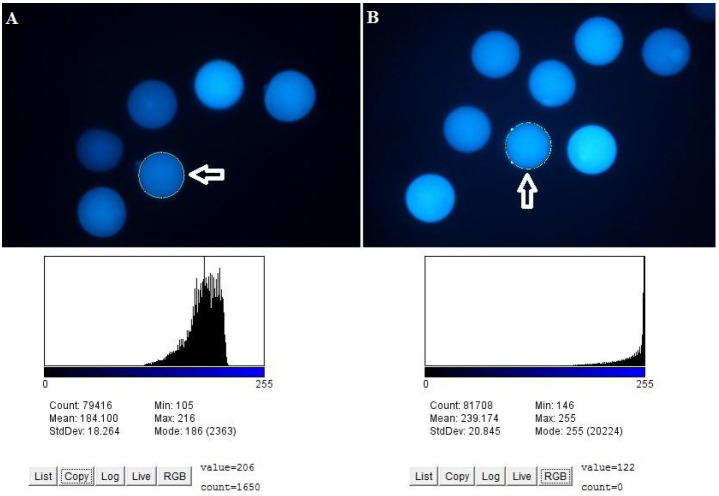
Representative images of oocytes’ fluorescent intensity for glutathione content.

Glutathione content measured by fluorescent intensity of mature oocytes was enhanced (P < 0.05) when royal jelly was supplemented to IVM medium at 10 mg/ml (293.4 arbitrary units), 5 mg/ml (286.5 arbitrary units), and 2.5 mg/ml (272.8 arbitrary units) compared with the control (210.6 arbitrary units) group ( Fig. 2 and 3 ).

### Early development and blastocyst rate

The rate of cleaved and day 8 embryos was higher (P < 0.05) in the royal jelly-treated groups at a concentration of 5 mg/ml (70.2% ± 3.2 and 33.1% ± 2.2) than in 2.5 mg/ml (59.2% ± 3.3 and 21.2% ± 4.1) and the control groups (54.5% ± 3.6 and 22.3% ± 3.7). Compared to other experimental groups, no significant (P <  0.05) improvement was observed in the percentage of cleaved embryos (69.8% ± 2.1) and day 8 blastocysts (26.4% ± 3.5) when royal jelly was added at a concentration of 10 mg/ml ( Table 3 ).

**Table 3:** Embryo development of royal jelly-treated and control oocytes at 8 days after *in vitro* fertilization (IVF) in goats (cumulative results of five replicates).

		Embryo development (mean ± SEM)	
Groups	Oocytes (n)	Cleaved (%)	Blastocyst (%)
Control	112	54.5 ± 3.6^b^	22.3 ± 3.7^b^
RJ 2.5	121	59.2 ± 3.3^b^	21.2 ± 4.1^b^
RJ 5.0	118	70.2 ± 3.2^a^	33.1 ± 2.2^a^
RJ 10.0	107	69.8 ± 2.1^a^	26.4 ± 3.5^ab^

RJ 2.5: Control maturation medium supplemented with 2.5 mg/ml royal jelly; RJ 5.0: Control maturation medium supplemented with 5.0 mg/ml royal jelly; RJ 10.0: Control maturation medium supplemented with 10.0 mg/ml royal jelly. ^a,b^ Within columns, values with uncommon superscripts differ (P < 0.05).

### Total number of cells and the number of apoptotic cells per blastocyst

Blastocyst cell count was similar (P < 0.05) in the control (114.7 ± 8.0) and treated groups at a concentration of 5 mg/ml (117.5 ± 9.6; Table 4 ). However, the number of apoptotic cells was higher (P < 0.05) in the control (7.9 ± 2.2) than in the treated group (4.1 ± 1.4).

**Table 4:** Total cell number and apoptotic cell number in blastocysts derived from royal jelly-treated and control oocytes.

			Embryo quality (mean ± SEM)	
Groups	Blastocysts (n)		Blastocyst nuclei (n)	Apoptotic cells (n)
Control	23		114.7 ± 8.0	7.9 ± 2.2^a^
RJ 5.0	26		117.5 ± 9.6	4.1 ± 1.4^b^

RJ 5.0: Control maturation medium supplemented with 5.0 mg/mL royal jelly. ^a,b ^Within columns, values with uncommon superscripts differ (P < 0.05).

### Gene expression profile

**Figure 4:**
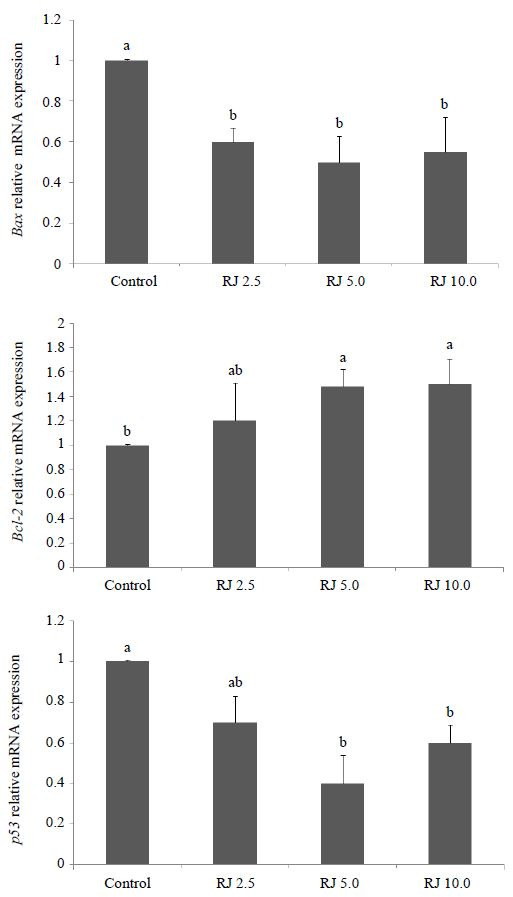
Expression profile of *Bax*, *Bcl-2*, and *p53* genes in oocytes treated with different concentrations of royal jelly during IVM using quantitative real-time PCR. ^a,b,c,d^Bars with uncommon superscripts are different (P < 0.05).

The expression profile of apoptotic-induced (*Bax* and *p53* ) and an anti-apoptotic (*Bcl-2*) gene were performed in oocytes after IVM in the presence or absence of royal jelly ( Fig. 4 ). Both genes involved in apoptosis induction (*Bax* and *p53* ) were expressed more highly (P <  0.05) in control oocytes compared to the royal jelly-treated ones at concentrations of 5 and 10 mg/ml. However, when royal jelly was added to IVM medium at 2.5 mg/ml, the expression profile of *p53* was not different (P > 0.05) from either the control or other treatment groups. On the other hand, expression of anti-apoptotic *Bcl-2* transcript was increased significantly in all oocytes treated with royal jelly at concentrations of 5 and 10 mg/ml, while at 2.5 mg/ml it was expressed in treated oocytes similarly to all other experimental groups ( Fig. 4 ).

## Discussion

The oocyte microenvironment is a key determinant factor in IVEP success ( Greve *et al.,* 1987 ; Rizos *et al.,* 2002 ; Sutton *et al.,* 2003 ). There are different ways to improve the oocyte microenvironment during the course of the maturation process. Co-culture of intact COCs with a specific ratio of denuded oocytes has improved nuclear, cytoplasmic maturation ( Dey *et al.,* 2012 ; Souza-Fabjan *et al.,* 2016 ) and the rate of blastocyst formation, in addition to quality ( Choi *et al.,* 2013 ). Antioxidants such as peroxiredoxin II ( Fakruzzaman *et al.,* 2015 ), melatonin ( Do *et al.,* 2015 ), resveratrol ( Kwak *et al.,* 2012 ), l-carnitine ( Mishra *et al.,* 2016 ), cysteine ( Ali *et al.,* 2003 ), and cysteamine ( Rodríguez‐González *et al.,* 2003 ) have been supplemented to IVM media to improve developmental competence of pre-implantation blastocysts of different mammalian species. In our study, supplementation of royal jelly to IVM medium has increased the rate at which oocytes reached MII-stage in a dose-dependent manner, although the cumulus expansion was not different from that of the control group. In agreement with our results, Valiollahpoor *et al*. (2016) has reported higher maturation rates of sheep oocytes when cultured in the presence of royal jelly at various concentrations. The positive effect of royal jelly in improving COC maturation could be due to the various active compounds like proteins, essential amino acids (cystine, lysine and arginine), sugars (fructose, glucose, and sucrose), vitamins (A, B5, C, D and E), and lipids ( Howe *et al.,* 1985 ; Boselli *et al.,* 2003 ; Kodai *et al.,* 2007 ; Tamura *et al.,* 2009 ). An early study observed increased errors in chromosomal segregation due to high oxidative stress during IVM, which compromises oocyte developmental potential after IVF in mice ( Tarin *et al.,* 1996 ).

Intracellular content of GSH is one of the crucial indicators of the developmental capacity of COCs. Highly developmental competent goat BCB+ oocytes have previously been shown to increase the intracellular GSH content compared with BCB- (with low developmental competence) and control oocytes ( Abazari-Kia *et al.,* 2014 ). The results of the present study indicated enhancement of oocytes glutathione content linearly with increasing concentrations of royal jelly supplemented to IVM medium, which subsequently increased maturation rate. The content of GSH and ATP concentrations has been analyzed in *in vitro-* and *in vivo*-matured porcine COCs ( Brad *et al.,* 2003 ). Porcine oocytes matured *in vivo* had a significantly higher content of GSH and developmental competence compared with *in vitro*-matured counterparts ( Brad *et al.,* 2003 ). Lower intracellular GSH was also linked with reduced developmental potential of bovine oocytes ( Hashimoto *et al.,* 2000 ; Furnus *et al.,* 2008 ). The enhanced effect of royal jelly on intracellular GSH content could be due to an increase in the synthesizing of GSH within COCs during IVM. In accordance with our results, supplementation of IVM media with crocin as an antioxidant substance has increased nuclear maturation rate and subsequent developmental potential of mouse oocytes, which may occur through its beneficial effect in increasing GSH concentrations in MII oocytes ( Maleki *et al.,* 2016 ). Similarly, IVM media supplemented with an antioxidant known as either resveratrol ( Kwak *et al.,* 2012 ) or carboxyethyl germanium sesquioxide (Ge-132; Kim *et al.,* 2015 ) improved embryonic development after parthenogenetic activation of porcine COCs and SCNT ( Jin *et al.,* 2016 ) by increasing the intracellular GSH levels.

These findings also highlight a vital biological property of royal jelly as an antioxidant ( Bărnuţiu *et al.,* 2011 ) that protects female and male gametes ( Guo *et al.,* 2009 ) and cryopreserved semen ( Moradi *et al.,* 2013 ; Shahzad *et al.,* 2016 ). In support of the antioxidant property, expression profile of apoptosis-related transcripts (*Bax* and *p53*) was suppressed in oocytes treated with royal jelly at 5 and 10 mg/ml. Additionally, the transcript abundance of anti-apoptotic gene (*Bcl-2*) was up-regulated after supplementation of IVM medium with royal jelly of 5 and 10 mg/ml. The antioxidative activity of royal jelly has been confirmed through its protection against oxidative stress when given to experimental animals ( El-Nekeety *et al.,* 2007 ; Silici *et al.,* 2009 ). Moreover, growing rabbits administered royal jelly have improved their physiological conditions ( Elnagar *et al.,* 2010 ), while adult males have experienced reduced summer infertility under heat stress ( Elnagar, 2010 ). Also, pregnancy rate and overall reproductive performance was improved in ewes administered royal jelly ( Kridli *et al.,* 2003 ; Husein and Haddad, 2006 ; Kridli and Al-Khetib, 2006 ). Thus, results from this and previous studies confirm the beneficial antioxidative effect of royal jelly in reducing the incidence of apoptosis in oocytes during IVM, which was coupled with enhancing mitochondria activity.

The influence of royal jelly in improving oocyte maturation and quality was extended to improve the percentage of cleaved embryos and blastocyst rate in the royal jelly-treated groups at concentrations of 5 and 10 mg/ml compared to 2.5 mg/ml and the control group. The synergistic effect of royal jelly on embryo development could be attributed to improving the IVM microenvironment ( Choi *et al.,* 2013 ), in addition to increasing nuclear and cytoplasmic maturation of oocytes ( Dey *et al.,* 2012 ). Interestingly, it seems that the royal jelly antioxidative potentiality could also be sustained throughout embryo cleavage until blastocyst formation, as observed in the current study and previous reports when various antioxidant supplements were used during IVM ( Ali *et al.,* 2003 ; Rodríguez‐González *et al.,* 2003 ; Kwak *et al.,* 2012 ; Do *et al.,* 2015 ; Fakruzzaman *et al.,* 2015 ; Mishra *et al.,* 2016 ). Enhancement of bovine oocytes’ glutathione content was induced by supplementation of IVM media with antioxidants (cysteamine, cysteine, and mercaptoethanol), and improved results of IVEP ( de Matos *et al*., 1995 , 1996 ). The induction of high GSH levels during IVM was continued to the beginning of embryo culture ( de Matos and Furnus, 2000 ). The increased level of GSH was coupled with reducing the intracellular levels of ROS, oxidative stress-induced apoptosis, and altering the expression of oocytes’ molecular markers during IVM ( Kwak *et al.,* 2012 ; Kim *et al.,* 2015 ; Jin *et al.,* 2016 ). In support to this assumption, addition of resveratrol during IVM of goat COCs has significantly reduced both intracellular ROS and expression of proapoptotic (Bax) gene while increasing GSH levels ( Mukherjee *et al*., 2014 ).

Overall, improvement in oocyte quality as a result of royal jelly treatment was also reflected in reducing incidence of apoptosis in embryos. Addition of different antioxidants during IVM ( Leyens *et al.,* 2004 ; Fakruzzaman *et al.,* 2015 ) or IVC ( Ghanem *et al.,* 2014 ) has reduced the number of apoptotic cells in blastocysts.

Indeed, a higher incidence of apoptosis could result in fragmentation arrest of embryos ( Yoneda *et al.,* 2004 ; Favetta *et al.,* 2007 ), while embryos with a greater number of cells and lower incidence of apoptosis are more likely to survive and to develop to term after transfer to recipients ( Van Soom *et al.,* 2007 ). Supplementation of maturation medium with 5 mg/mL royal jelly increased the rate of embryo cleavage and blastocyst formation. This increase was associated with improvement of the nuclear maturation rate and cytoplasmic maturation, as shown by enhancing glutathione content and changing the expression of oocyte key genes to support subsequent embryo development. The apoptotic process was reduced in embryos developed from COCs treated with royal jelly. Therefore, our study confirms, at gene level, the beneficial antioxidant capacity of royal jelly, which could be used as a supplement of a promising IVM medium culture to enhance both embryo development and quality.
